# Food Allergies: Current and Future Treatments

**DOI:** 10.3390/medicina55050120

**Published:** 2019-05-01

**Authors:** Amelia Licari, Sara Manti, Alessia Marseglia, Ilaria Brambilla, Martina Votto, Riccardo Castagnoli, Salvatore Leonardi, Gian Luigi Marseglia

**Affiliations:** 1 Pediatric Clinic, Department of Pediatrics, Fondazione IRCCS Policlinico San Matteo, University of Pavia, 27100 Pavia, Italy; amelia.licari@unipv.it (A.L.); i.brambilla@smatteo.pv.it (I.B.); martinavotto@gmail.com (M.V.); riccardo.castagnoli@yahoo.it (R.C.); 2 Department of Pediatrics, Unit of Pediatric Genetics and Immunology, University of Messina, 98122 Messina, Italy; saramanti@hotmail.it; 3 Department of Clinical and Experimental Medicine, University of Catania, 95131 Catania, Italy; leonardi@unict.it; 4 Department of Pediatrics, University of Pavia, 27100 Pavia, Italy; alessiamarseglia@gmail.com

**Keywords:** allergen-specific therapy, allergen-nonspecific therapy, food allergy, biologics, children

## Abstract

Food allergies are an increasingly public health problem, affecting up to 10% of children and causing a significant burden on affected patients, resulting in dietary restrictions, fear of accidental ingestion and related risk of severe reactions, as well as a reduced quality of life. Currently, there is no specific cure for a food allergy, so the only available management is limited to strict dietary avoidance, education on prompt recognition of symptoms, and emergency treatment of adverse reactions. Several allergen specific- and nonspecific-therapies, aiming to acquire a persistent food tolerance, are under investigation as potential treatments; however, to date, only immunotherapy has been identified as the most promising therapeutic approach for food allergy treatment. The aim of this review is to provide an updated overview on changes in the treatment landscape for food allergies.

## 1. Introduction

Food allergies (FA) are defined as an adverse health effect arising from a specific immune response that occurs, reproducibly, on exposure to a given food [1]. The immune response to food may be immunoglobulin E (IgE)-mediated (immediate reactions), non-IgE mediated (delayed reactions) or mixed. Recent data suggest that IgE-mediated food allergies are common, affecting up to 10% of children [2] with increasing prevalence in the last decades [3,4,5]. Food allergies pose a significant burden on affected children and their families, resulting in dietary and social restrictions, fear of accidental reactions, high levels of anxiety related to risk of severe reactions, fatalities and, as a consequence, a reduced quality of life [6]. While some food allergies (milk, egg, wheat, and soy) typically have a high rate of resolution in childhood and adolescence, others, such as peanut, tree nut, fish and shellfish allergies, tend to be lifelong or rarely resolved. With the absence of a definitive cure, current effective management of an IgE-mediated food allergy is based on patient and family education, strict allergen avoidance, and prompt recognition and treatment of allergic reactions. However, diet adherence and self-management of anaphylactic reactions show low compliance, especially in adolescents [7]. Recently, more active approaches to the management of food allergies have emerged. Such approaches include early dietary introduction of potentially allergenic foods and immunotherapy (Table 1), which aim to prevent the development of FA and to restore or induce immune tolerance against food allergens, respectively. The aim of this review is to focus on current and future treatment approaches of FA in children, with particular reference to immunotherapy, which has recently shown promising results with its potential to be disease modifying.

## 2. Methods

### 2.1. Research Strategy

This review has been conducted employing PubMed and Science Direct databases. On these websites, we searched for articles from 1 January 1997 to March 2019 using key terms related to food allergies: “food allergy”, “therapy”, “treatment”, “children”, and “adult”.

### 2.2. Study Selection

Articles were included in the review according to the following inclusion criteria: English language, publication in peer-reviewed journals, and year of publication at least 1997. Editorial, commentary, case report, and case series were excluded from the analysis.

Articles were excluded by title, abstract, or full text for irrelevance to the investigated issue. Lastly, to identify further studies that met the inclusion criteria, the references of the selected articles were also reviewed.

## 3. Allergen Specific Immunotherapy

Food allergy immunotherapy (FA-AIT) in all forms involves exposing the allergic subject to gradually increased doses of the allergenic food. Ideally, this active treatment enables an increase in the amount of food that the patient can intake without reacting during treatment (i.e., desensitization), reducing the risk of potential life-threatening allergic reactions in cases of accidental ingestion [8]. However, a more comprehensive goal of FA-AIT is the absence of symptoms after the intake of a normal serving of the culprit food despite a period of absence of exposure (i.e., tolerance), thus maintaining its efficacy after the discontinuation of the treatment [9]. The achievement of a persistent tolerance is still one of the unmet needs of FA-AIT, and it is currently recommended to consume the allergenic food in order to maintain the beneficial effects of desensitization [10].

The most frequently studied food allergens are cow’s milk, hen’s eggs and peanuts, although trials have been carried out on peaches, hazelnuts, wheat and kiwifruit, with some investigations performed using multiple foods [11].

The routes of administration of FA-AIT include: OIT (oral immunotherapy), SLIT (sublingual immunotherapy) and EPIT (epicutaneous immunotherapy) [12].

With OIT, the allergic patient swallows the food allergen and the total amount of food is gradually increased until it is tolerated at usual doses. Trials with OIT have demonstrated clinical efficacy and the possibility to achieve desensitization is estimated up to 90%; however, these promising results are hampered by adverse events due to the direct intake of the culprit food [13]. So far, due to the lack of standardized OIT products, fresh foods have mainly been used. Recently, results from the first multicenter, randomized, placebo-controlled, phase three trial on a newly-developed oral product containing a well-characterized peanut protein profile met both the safety and effectiveness outcomes [14]. Moreover, in order to reduce the adverse events without affecting efficacy, gastro-intestinal delivery oral immunotherapy (GIDOIT) using peanuts in sealed capsules has been studied with encouraging results [15].

The European Academy of Allergy and Clinical Immunology (EAACI) recently developed clinical guidelines on AIT for IgE-mediated food allergies: Overall, OIT is actually recommended for persistent cow’s milk, hen’s egg, or peanut allergies for children around 4 to 5 years of age on the basis of its ability to increase the threshold for clinical reactions while on OIT (grade A of recommendation) [13].

In SLIT, patients are treated with drops of food allergen extract that are placed under the tongue. SLIT dosage is lower than OIT and limited to the concentration of available extracts, thus reducing the risk of adverse reactions. However, the rate of successful desensitization is reduced compared to OIT [13].

In EPIT, an allergen-containing patch is applied to the skin in order to facilitate the absorption of the food protein allergen by dendritic cells in the dermis that, once migrated to local draining lymph nodes, present the antigen and promote the generation of regulatory T (T-reg) cells [16]. Promising preclinical studies in animals paved the way for the clinical development of peanut EPIT [17]. A multicenter, double-blind, randomized, placebo-controlled study, conducted in 74 peanut allergic subjects (aged 4–25 years) showed an optimal safety profile, with only local reactions at the application site and no instances of anaphylaxis; however, after 52 weeks, a modest short-term efficacy was reported, with the highest responses among younger children (4–11 years old) [18]. Results from an ongoing phase three clinical trial in peanut allergic children are awaited to better identify optimal candidates for treatment with EPIT and the ideal treatment duration [19].

Overall, FA-AIT is currently the only potentially curative treatment for immunoglobulin (Ig)E-mediated FA. Data from clinical trials are promising; however, there are still several unmet needs for clinical practice [13,20,21]. Regarding side effects, no fatalities have been described so far, but systemic reactions are reported as quite common [8]. In this context, in order to reduce the risk of adverse allergic reactions while on AIT, biologicals (e.g., omalizumab) have been investigated with encouraging results [22,23]. Moreover, anti-IgE monoclonal antibodies (omalizumab) could be an important therapeutic tool for treatment of patients with the most severe phenotype of life-threatening anaphylaxis and those with concomitant bronchial asthma, which represent the most difficult cases in clinical practice.

## 4. Allergen Nonspecific Immunotherapy

For patients affected by multiple and concomitant FA, allergen nonspecific therapies are strongly attractive. These approaches have cytokines, Toll-like receptors (TLRs), cells, IgE, probiotics, and genes as targets.

### 4.1. Anti-Cytokines Therapy

In light of evidence that cytokine signaling drives inflammatory responses, authors postulated that FA could be prevented by cytokine blocking agents [24]. In a murine model, the administration of *Lactococcus lactis*, transfected to secrete interleukin (IL)-10, provided protection from food-induced anaphylaxis [25]. Similar results were obtained after the administration of recombinant mouse IL-21 or an IL-21 expression plasmid in a mouse model strongly-sensitized to peanuts. Specifically, the anaphylactic reaction was abolished, as well as a significant decrease in the serum total and specific IgE levels [26]. In an ovalbumin food allergy murine model, oral administration of transforming growth factor-beta (TGF-β) allowed acquisition of ovalbumin tolerance, which was assessed by a decrease in ovalbumin specific IgE and IgG1 antibodies and T-cell reactivity, and confirmed by a reduction in the immediate-type skin reaction [27]. The anti-IL-33 antibody, ANB020 (AnaptysBio, San Diego, CA, USA), has shown satisfactory results in a phase two placebo-controlled clinical trial designed to investigate efficacy and safety in an adult population affected by peanut allergy [28]. No clinical trials have investigated the effectiveness of anti-cytokine therapies in the context of FA in children [29].

### 4.2. Toll-Like Receptors (TLRs)

Toll-like receptors (TLRs) are a class of receptors expressed on dendritic cells (DC) and macrophages that, upon activation of an immune response, enhance a tolerogenic response and restore the T helper (Th)1/Th2 balance in Th2-mediated allergic disorders [30]. TLRs may be stimulated by microbial particles (mainly lipopolysaccharide) as well as by specific agonists such as TLR7 agonist R848 [31], TLR9 agonist CpG oligodeoxynucleotides [32], and TLR4 agonist monophosphoryl lipid A [33]. Of these, only the TLR9 agonist has been investigated in a murine model of FA. Following oral administration, a decrease in gastrointestinal inflammation, a reduction in levels of peanut-specific IgE and an increase in IgG2 values, as well as protection from peanut-anaphylaxis, were observed [34]. No data are available in humans affected by FA.

### 4.3. Cellular Targets

Even if no specific cell therapeutic targets have been developed for the treatment of FA, there are numerous candidates for future therapies. Intestinal epithelial cells (IEC), DC and T-reg cells showed positive effects in triggering immune tolerance, inhibiting IgE transport, and reducing Th2-driven inflammation [35].

### 4.4. Anti-IgE Therapy

The first investigation of anti-IgE therapy for the management of FA was performed in a double-blind, randomized, dose-ranging (150, 300, or 450 mg of anti-IgE antibodies (TNX-901)) trial in 84 patients, 12 to 60 years of age, with a positive history of peanut allergy. Although the highest TNX-901 dose significantly improved clinical symptoms and increased the threshold dose for peanuts, 25% failed to develop a tolerance to peanuts, suggesting a wide treatment response variability [36]. A subsequent double-blinded, placebo-controlled study was started in children 6 years of age, but discontinued because of safety issues related to pre-omalizumab challenges [37]. An open-label study in 14 adults between 18 and 50 years of age showed a significant increase in the mean tolerated dose of peanut protein (from 80 mg to 5080 mg) after 6 months of omalizumab; however, the administration of antihistamines and epinephrine was required in 10 of the 14 enrolled subjects [38]. To increase the safety of immunotherapy and possibly enhance tolerance development, a combination of anti-IgE therapy and FA-AIT was investigated. Two small double-blind, placebo-controlled food challenge trials in patients (age, 7–25 years) with a peanut [39] or cow’s milk [40] allergy were conducted by using omalizumab in combination with rapid oral food desensitization. During a washout period, participants were generally treated with omalizumab for 2 to 5 months and subsequently continued on treatment until a maintenance dose of OIT was achieved. In the first study, 92% of patients tolerated the challenge, but 46% of children experienced moderate to severe adverse events [39]. In the second trial, 9 out of 11 patients were able to complete dose escalation and only 1.8% of subjects still showed reactions requiring epinephrine [40]. Subsequently, a phase one clinical trial was designed in 25 participants (median age 7 years) with multiple FA. Participants were receiving OIT for up to 5 allergens simultaneously with omalizumab. Anti-IgE therapy was administered for 8 weeks prior to and 8 weeks following the initiation of the OIT protocol. Adverse reactions were reported in 5.3% of subjects. Additionally, 94% of reactions were mild and only one subject experienced a severe reaction requiring epinephrine [23]. Following this, a phase one double-blind, placebo-controlled food challenges study, enrolling patients aged 4–15 years with multiple FA, confirmed that adjunctive omalizumab with OIT provided a safe and rapid desensitization with a lower median rate of adverse events (27% vs. 68%). Interestingly, no serious or severe adverse events were recorded [41].

The determination of the specific role of omalizumab in tolerance development was first investigated in a double-blind placebo-controlled trial comparing omalizumab with a placebo as an adjunctive therapy for cow’s milk OIT in 57 subjects (7–32 years) with severe cow’s milk allergy. During a washout period, participants received 4 months of omalizumab and were subsequently continued on treatment until a maintenance dose of OIT was achieved (at 28 months). Although no differences were detected in the rates of desensitization, significantly fewer reactions requiring epinephrine occurred in the omalizumab-treated group as compared to the placebo-treated group (2 vs. 18 doses) [22]. These findings were confirmed in a subsequent case series on 14 egg-allergic and cow’s milk–allergic children (age, 4 months to 11 years). All patients were able to tolerate OIT only when omalizumab was administered as a pretreatment and in conjunction with OIT [42]. Lastly, in a post-hoc analysis, Bedoret et al. postulated that an anergy of the milk-specific CD4-T cells could be implicated in omalizumab-mediated allergen desensitization [43]. Taken together, these data suggest the possibility of using omalizumab as a therapeutic weapon to increase threshold tolerance levels, providing more protection in cases of accidental ingestion in patients with FA [39,40,41,42,43,44]. However, to date, omalizumab is still an off-label treatment with no established dosages. Recently, an individualized anti-IgE treatment, both in terms of dose and length, has been proposed through monitoring of basophil allergen threshold sensitivity [45]. To fill the gap in the evidence supporting omalizumab as a monotherapy or in combination with OIT for food allergy treatment, a clinical development plan is currently ongoing (Table 2).

### 4.5. Probiotics

The use of probiotics in the prevention or treatment of FA is based on the concept of colonizing the gastro-intestinal tract with health-promoting organisms with positive benefits. Immune-modulation, competitive exclusion, and release of gut mucin secretion, as well as the production of compounds inhibiting the growth of other bacteria have been postulated as mechanisms of action for probiotics [46]. Following encouraging findings from experimental models [47,48], several studies have been designed to examine the efficacy of probiotics in the prevention and/or treatment of FA in humans [49].

To investigate the effect of probiotics on the prevention of FA, a double-blind, placebo-controlled trial was performed on pregnant mothers who were either receiving *Lactobacillus GG* (LGG) or a placebo during the last 4 weeks of pregnancy and during subsequent breastfeeding until the infant reached 3 months of age. When compared to the control group, the probiotic group showed significantly higher serum TGF-β2 levels and a lower incidence in atopic eczema [50]. However, these findings were not replicated in a 4-year follow-up of a randomized placebo-controlled trial, in which both prenatal and postnatal supplementation failed to show any effect on IgE sensitization to food or environmental allergens [51]. Overall, a systematic review and meta-analysis by Zhang et al., evaluating the results of 17 trials involving 2947 infants, concluded that when administered prenatally to the pregnant mother and postnatally to the child, probiotics significantly reduced the risk of atopy (relative risk (RR) 0.78; 95% confidence interval (CI) 0.66–0.92; I2 = 0%). No effects on atopy and food hypersensitivity were recorded when probiotics were administered either prenatally or postnatally [52].

With regard to the efficacy of probiotics in food allergy treatment, clinical trials of probiotic supplementation with LGG, combined with extensively hydrolyzed casein formula in milk-allergic children, demonstrated increased rates of milk allergy resolution after 1 [53], 6 [54] and 12 months [55], compared with a control group receiving the formula alone. At follow-up at 1 month, fecal eosinophil cationic protein and tumor necrosis factor-alpha (TNF-a) were significantly decreased in children receiving LGG in their extensively hydrolyzed formula [53]. Also, a clinical resolution was recorded at 6 and 12 months follow-up in the experimental arm compared with control group [54]. However, no differences in the cumulative percentage of tolerance to cow’s milk were reported among groups at 12 months [55]. As the benefits of probiotics were thought to result from their ability to restore the natural balance of gut bacteria, Berni et al. [56] tested this hypothesis by comparing stool from cow’s milk allergic children to that from healthy infants before and after treatment with extensively hydrolyzed formula with or without LGG. The authors noted that the gut microbiome of infants which achieved the immune tolerance was enriched in *Blautia* and *Roseburia* and possessed higher concentrations of the short-chain fatty acid butyrate. This led the researchers to hypothesize that probiotics, through modulation of the host–gut ecosystem and, consequently, the local metabolism, work positively to favor the acquisition of ‘tolerance-associated’ microbial profiles [56]. Recently, authors evaluated the baseline presence of *Bifidobacterium longum* BB536 (BL), *Bifidobacterium breve* M-16V (BB) and *Bifidobacterium infantis* M-63 (BI) in children, aged 10–14 months, with an IgE-mediated cow’s milk allergy before, during, and after administration of multi-strain probiotics containing 3.53109 UFC of BL, BB and BI. Following probiotics administration, a significant increase in BI concentration was observed, demonstrating the health-promoting effects of probiotics [57].

The rationale for an effect of probiotics on other FA has also been translated on other food allergens, including peanut allergy. The effect of probiotics as an adjuvant to OIT has been evaluated in a double-blind placebo-controlled randomized trial involving a pediatric population (1–10 years) affected by peanut allergy. Co-administration of *L. rhamnosus* CGMCC1.3724 and peanuts led to sustained desensitization and reduced serum specific IgE levels [58]. These positive effects were maintained over time. A follow-up study 4 years after treatment cessation reported that participants from the probiotic and peanut OIT (PPOIT) group were significantly more likely than those from the placebo group to have continued eating peanuts (*p* = 0.001), also showing smaller wheals in peanut skin prick tests and significantly higher peanut serum (s)IgG4:sIgE ratios when compared to the placebo [58]. However, due to the lack of individuals in the OIT-only or probiotic-only group, the efficacy attributable to the probiotic remains unclear.

The evidence for preventive and therapeutic effects of probiotics on FA in human subjects is still sparse [59,60]. More data are needed to support probiotic supplementation for FA. Regarding the instances where a reduction in clinical symptoms in infants was reported, the effects were not consistent between studies and caution is advised due to methodological aspects, excess losses in patient follow-up, and substantial heterogeneity among included studies in regard to type of strains, duration of treatment, and doses administered [61].

### 4.6. Gene Therapy

As the poor treatment persistence of biologics can blunt the effectiveness, a monthly parenteral administration of the omalizumab is suggested. Widely used for gene transfer, adeno-associated virus (AAV) vectors are very attractive for treatment of FA as they ensure a persistent release of anti-human IgE, guaranteeing protection over time. Accordingly, to avoid repeated drug administrations, researchers have hypothesized that the administration of an AAV coding for omalizumab could provide long-lasting protection against food-induced allergic reactions [62]. This hypothesis was tested using a humanized murine model of peanut allergy and revealed that a single administration of a AAVrh.10anti-hIgE vector ensured protection from food-induced anaphylaxis through a sustained and continuous release of anti-human IgE. Importantly, data showed that a single administration protected the humanized murine model from FA, and the treatment also appeared efficacious both before and after peanut sensitization. Moreover, not being a therapy targeted against a specific allergen, the efficacy of AAVrh.10 anti-human IgE treatment seems potentially transferable and applicable to other food allergens, ushering in a new era for food allergy control and treatment.

## 5. Conclusions

The rising incidence of FA requires increasingly effective and safe therapeutic strategies. Biologics represent a new treatment option to influence the mechanisms underlying FA and to more rapidly reach the immune tolerance for food antigens. Immunotherapy clinical trials show encouraging results, with an acceptable efficacy profile. However, numerous mild to severe adverse reactions can and have occurred during treatment, and thus current protocols have suggested and tested the use of anti-IgE antibodies as an adjunctive therapy with OIT, showing a satisfactory safety profile. Nevertheless, the optimal dosage, duration of treatment and long-term effects of biologicals as a monotherapy or in combination with OIT remain to be elucidated. Recent experimental studies have identified other non-allergen-specific molecules as potential targets for management of patients with multiple food allergies. Cytokines, TLRs, cells, probiotics, and genes are currently being investigated, but their use in humans is still far from clinical application at this time.

## Figures and Tables

**Table 1:** List of licensed and potential strategies for treating food allergies.

Therapy	Mechanism of Action	Population	Status
Allergen specific			
Allergy immunotherapy OIT * SLIT * EPIT *	Prolonged exposure to antigen restores the Th1/Th2 * balance, promoting Treg * activity	Pediatric Adult	Clinical trials (Phase 3)
Allergen non specific			
Cytokines	Influence with inflammatory pathways	NA *	Murine models Clinical trials (Phase 2)
Toll Like receptors	Activate the immune response Enhance the tolerogenic response Restore the Th1/Th2 balance	NA	Murine models
Cellular target	Trigger immune tolerance Inhibition IgE transport Reduction in Th2-driven inflammation	NA	Murine models
Anti-IgE	Inactivaction IgE * Prevention of stimulation of high affinity IgE-receptor	Pediatric Adult	Clinical trials (Phase 2)
Anti-IgE with OIT	Improve efficacy OIT Improve safety OIT	Pediatric Adult	Clinical trials (Phase 2)
Probiotics	Immune-modulation Competitive exclusion Release of gut mucin secretion Production of compounds inhibiting the growth of other bacteria	Pediatric Adult	Clinical trials
Gene therapy	Persistent release of anti-human IgE	NA	Murine models

* OIT: oral immunotherapy; SLIT: sublingual immunotherapy; EPIT: epicutaneous immunotherapy; Th1: lymphocytes T helper 1; Th2: lymphocytes T helper 2; Treg: Regulatory T; NA: not applicable; IgE: immunoglobulin E.

**Table 2:** Clinical development program for biologicals as monotherapies or as adjunctive treatments with immunotherapy in food allergy treatment.

	Clinical Trial Identifier	Study Title	Status	Phase	Estimated Enrollment (N. pts) *	Ages Eligible for Study	Primary Outcome Measures	Interventions	Drug Dosage	Preliminary Results
1	NCT02879006	E-B-FAHF-2, Multi OIT * and Omalizumab for Food Allergy	Recruiting	2	34	6 to 40 y *	Sustained unresponsiveness	Chinese Herbal Medication Placebo Omalizumab Multi OIT	Not applicable	Not applicable
2	NCT02643862	Study Using Omalizumab in Rush Multi Oral Immunotherapy in Multi Food Allergic Patients (MAP-X)	Completed	2	48	4 to 55 y	Desensitization measured by proportion of FA * participants who pass a DBPCFC * to 2000 mg protein for each of 2 allergens at week 36	Omalizumab Placebo	Not applicable	Not applicable
3	NCT03181009	Multi OIT to Test Immune Markers After Minimum Maintenance Dose	Recruiting	2	60	2 to 25 y	Change in allergen-specific serum IgG4 * and IgE	Omalizumab Food Flour Allergens	Omalizumab: subjects ≥ 4 yrs receive 150 mg *. Subjects ≤ 4 yrs receive 75 mg Food Flour Allergens: 300 to 1200 mg	Not applicable
4	NCT02626611	Multi Immunotherapy to Test Tolerance and Omalizumab	Completed	2	70	4 to 55 y	The number of participants able to tolerate an oral food challenge to 2000 mg at least of 2 allergens at week 36	Omalizumab Food Flour Buildup	Omalizumab: not applicable Food Flour Buildup: up to 2000 mg	Not applicable
5	NCT01510626	Omalizumab With Oral Food Immunotherapy With Food Allergies Open Label Safety Study in a Single Center	Completed	1	35	4 to 55 y	Number of adverse events in the treatment population	Omalizumab Food protein	Not applicable	Not applicable
6	NCT00949078	Omalizumab in the Treatment of Peanut Allergy	Completed	2	51	18 to 50 y	Number of pts who experienced a decrease in Pn-BHR * AUC * of >80% compared with baseline values before week 8 percent change in peanut specific IgE from baseline to after Pn-BHR response percentage peanut specific IgE after pn-BHR response total IgE after pn-BHR response Dose of peanut protein inducing allergic symptoms at OFC1 * Dose of peanut protein inducing allergic symptoms at OFC 2 Dose of peanut protein inducing allergic symptoms at OFC 3 Omalizumab received before OFC 2 number of doses omalizumab received before OFC 2	Omalizumab Food allergen	Not applicable	Not applicable
7	NCT01781637	Peanut Reactivity Reduced by Oral Tolerance in an Anti-IgE Clinical Trial	Active, not yet recruiting	1, 2	36	7 to 25 y	Tolerance of 2000 mg 6 weeks after last dose of omalizumab/placebo	Omalizumb Placebo	Not applicable	Not applicable
8	NCT03881696	Omalizumab as Monotherapy and as Adjunct Therapy to Multi-Allergen OIT in Food Allergic Participants	Not yet recruiting	3	225	2 to 55 y	Number of participants by stage 1 treatment group, omalizumab versus placebo, who successfully consume ≥600 mg of peanut protein without dose-limiting symptoms during the DBPCFC conducted at the end of treatment stage 1	Omalizumab Placebo Multi-Allergen Oral Immunotherapy	Omalizumab: 75 to 150 mg	Not applicable
9	NCT02402231	Treatment of Severe Peanut Allergy With Omalizumab and Oral Immunotherapy (FASTX)	Active, not recruiting	2	23	12 to 22 y	Peanut challenge	Omalizumab Immunotherapy	Not applicable	Not applicable
10	NCT01157117	OIT and Omalizumab in Cow’s Milk Allergy	Completed	2	77	7 to 35 y	Percentage of Subjects in the Omalizumab Group vs. Placebo Group Developing Clinical Tolerance to Milk	Omalizumab Milk powder	Omalizumab: not applicable: Milk powder: up to 3.84 g *	Omalizumab vs * Milk powder: *p* = 0.42
11	NCT03679676	Clinical Study Using Biologics to Improve Multi OIT Outcomes	Not yet recruiting	2	200	6 to 21 y	Successful food challenges to two or more FA at week 38 between cohort omalizumab and cohort placebo	Omalizumab Placebo Dupilumab	Not applicable	Not applicable
12	NCT00968110	Omalizumab Treatment for Milk Allergic Children	Completed	1	10	4 to 18 y	The major goal of this study is to assess the safety of Omalizumab in young children, and the safety of oral desensitization in patients pretreated with Omalizumab	Omalizumab	Not applicable	Not applicable
13	NCT00086606	A Safety and Efficacy Study of Omalizumab in Peanut Allergy	Terminated	2	150	6 to 75 y	Not applicable	Omalizumab	Not applicable	Not applicable
14	NCT00932282	Peanut Oral Immunotherapy and Anti-IgE for Peanut Allergy (PAIE/Omalizumab)	Terminated	1, 2	13	12 y and older	The percentage of subjects who pass the 20 mg peanut flour (~50% peanut protein) OFC 2–4 weeks after discontinuing peanut OIT therapy	Peanut Oral Immunotherapy Omalizumab	Peanut Oral Immunotherapy: 0.2 mg of peanut flour to 8000 mg Omalizumab: not applicable	Not applicable
15	NCT00382148	A Study of Omalizumab in Peanut-Allergic Subjects Previously Enrolled in Study Q2788g	Completed	2	10	6 to 75 y	Serious Adverse Events	Omalizumab	Not applicable	Not applicable
16		Omalizumab Enhances Oral Desensitization in Peanut Allergic Patients	Completed	1, 2	13	7 to 25 y	Number of participants that tolerated rapid oral peanut desensitization to a dose of 500 mg peanut flour	Omalizumab	Not applicable	Not applicable

* OIT: oral immunotherapy; pts: patients; yrs: years; FA: food allergy; DBPCFC: Double-blind, placebo-controlled food challenge; Ig: immunoglobulin; mg: milligrams; Pn-BHR: peanut allergen induced basophil histamine release; AUC: Area under the curve; OFC: oral food challenge; g: grams; vs.: versus; y: years.
